# Prosthetic joint infection development of an evidence-based diagnostic algorithm

**DOI:** 10.1186/s40001-017-0245-1

**Published:** 2017-03-09

**Authors:** Heinrich M. L. Mühlhofer, Florian Pohlig, Karl-Georg Kanz, Ulrich Lenze, Florian Lenze, Andreas Toepfer, Sarah Kelch, Norbert Harrasser, Rüdiger von Eisenhart-Rothe, Johannes Schauwecker

**Affiliations:** 1 Department of Orthopaedic Surgery, Klinikum rechts der Isar, Technische Universität München, Ismaninger Str. 22, 81675 Munich, Germany; 2 Department of Trauma Surgery, Klinikum rechts der Isar, Technische Universität München, Ismaninger Str. 22, 81675 Munich, Germany

**Keywords:** Prosthetic joint infection, Algorithm, Total joint replacement, Revision surgery, THA, TKA

## Abstract

**Background:**

Increasing rates of prosthetic joint infection (PJI) have presented challenges for general practitioners, orthopedic surgeons and the health care system in the recent years. The diagnosis of PJI is complex; multiple diagnostic tools are used in the attempt to correctly diagnose PJI. Evidence-based algorithms can help to identify PJI using standardized diagnostic steps.

**Methods:**

We reviewed relevant publications between 1990 and 2015 using a systematic literature search in MEDLINE and PUBMED. The selected search results were then classified into levels of evidence. The keywords were prosthetic joint infection, biofilm, diagnosis, sonication, antibiotic treatment, implant-associated infection, *Staph. aureus*, rifampicin, implant retention, pcr, maldi-tof, serology, synovial fluid, c-reactive protein level, total hip arthroplasty (THA), total knee arthroplasty (TKA) and combinations of these terms.

**Results:**

From an initial 768 publications, 156 publications were stringently reviewed. Publications with class I–III recommendations (EAST) were considered. We developed an algorithm for the diagnostic approach to display the complex diagnosis of PJI in a clear and logically structured process according to ISO 5807.

**Conclusions:**

The evidence-based standardized algorithm combines modern clinical requirements and evidence-based treatment principles. The algorithm provides a detailed transparent standard operating procedure (SOP) for diagnosing PJI. Thus, consistently high, examiner-independent process quality is assured to meet the demands of modern quality management in PJI diagnosis.

## Background

The total number of hip and knee arthroplasties performed in the US is constantly increasing, with an expected increase in total knee arthroplasties (TKAs) of approximately 600% by 2030. Similarly, the number total hip arthroplasties (THAs) performed is estimated to triple during this period, leading to a significant increase in revision surgeries [1].

One major reason for revision arthroplasty is prosthetic joint infection (PJI). The incidence of PJI after primary surgery is 0.2–1.1%; in cases of revision surgery, it can reach 5% [2]. In contrast with acute PJI, low-grade infections are characterized by unspecific symptoms, such as pain and early implant loosening. Typical low-grade infections are often a result of infection with less virulent bacterial strains of the dermal flora, including coagulase-negative Staphylococci and Propionibacterium acnes, often lacking severe inflammatory symptoms [3].

Although it is of fundamental importance for further treatment, diagnosing a low-grade PJI prior to revision surgery can be challenging.

In addition to clinical findings, conventional radiographs, erythrocyte sedimentation rate (ESR), C-reactive protein (CRP), the percutaneous aspiration of synovial fluid for evaluating cell counts and differentials, and microbiology workups are routinely employed diagnostic tools (3).

Moreover, arthroscopically or fluoroscopically controlled biopsy of periprosthetic tissue can be performed. Some authors suggest the use of synovial biomarkers, leukocyte esterase tests or radionuclide imaging to support the diagnosis of a low-grade PJI [4, 5].

All tests have a role in the workup of PJI; however, the diagnostic values reported in the recent literature vary greatly [4–6].

To safely rule out or verify the presence of PJI prior to arthroplasty revision surgery, a customized combination of different diagnostic tests must be employed for every single case. Although the composition of an appropriate set of diagnostic tests may be self-evident for senior orthopedic surgeons who specialize in treating PJI, it can be challenging for attending orthopedic surgeons and general practitioners. The correct preoperative diagnosis is, however, of major significance because treatment strategies differ greatly between septic and aseptic revision surgery and have far-reaching consequences for the patient (20). Whereas false-positive findings lead to unnecessary two-stage revisions, one-stage revision surgery without the essential implementation of antibiotic therapy can result from false-negative results, inevitably causing new prosthetic failure and PJI persistence [6].

We, thus, developed an evidence-based diagnostic algorithm with examiner-independent diagnostic reliability for identifying PJI, and we prospectively observed its use in daily clinical routine according to the requirements of modern quality management in our institution.

## Methods

**Fig. 1:**
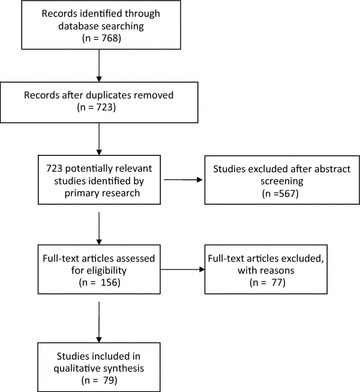
Flow chart of selection process

A systematic literature search was conducted in the databases of PubMed and Medline using the following search terms: prosthetic joint infection, implant-associated infection, biofilm, diagnosis, sonication, antibiotic treatment, microcalorimetry, *Staph. aureus*, coagulase-negative staphylococci, Propionibacterium rifampicin, implant retention, pcr, maldi-tof, serology, synovial fluid, C-reactive protein level, THA, TKA leucocyte esterase test, alpha-defensin test. All relevant publications between January 1990 and January 2015 were screened according to methodological aspects using QUADAS, STARD and PRISMA criteria [7–11] and classified according to the Grade system (The Grading of Recommendation Assessment, Development and Evaluation). Referring to the Grade system, the included studies were grouped according to the EAST classification, shown in Table 1 and Fig. 1, which allows the evaluation of medical publications, reviews and recommendations in terms of their level of evidence (LoE) and class of recommendation (CoR) [12–21].

**Table 1:** EAST level of evidence (LoE) and class of recommendation (CoR)

LoE	
I	Prospective randomized controlled trials (ORCTs)
II	Clinical studies in which the data was collected prospectively, and retrospective analyses which were based on clearly reliable data Types of studies so classified: observational studies, cohort studies, prevalence studies and case control studies
III	Studies based on retrospectively collected data. Evidence used in this class indicate clinical series, database or registry review, large series of case reviews and expert opinion
CoR	
I	The recommendation is convincingly justifiable based on the available scientific information alone. This recommendation is usually based on Class I data; however, strong Class II evidence may form the basis for a level I recommendation, especially if the issue does not lead itself to testing in a randomized trial.
II	The recommendation is reasonably justifiable by available scientific evidence and strongly supported by expert opinion This recommendation is usually supported by Class II data or a preponderance of Class II evidence
III	The recommendation is supported by available data but adequate scientific evidence is lacking This recommendation is generally supported by Class III data. This type of recommendation is useful for educational purposes and in guiding future clinical research

The literature review exclusively included studies of adults and publications in the English or German language. All references from the publications used in this study were examined for additionally relevant publications.

We identified 723 studies that met our search criteria. To perform statistically valid analyses, we only included studies with a minimum of 26 patients (Fig. 1).

Furthermore, only studies that used the definition criteria [22] of the Musculoskeletal Infection Society (MSIS) [23], Infectious Diseases Society of America (IDSA) [24] and International Consensus Meeting [25] (Table 2) were included.

**Table 2:** Definition of prosthetic joint infection

	MSIS		IDSA		International consensus	
	Main criteria	Supportive criteria	Main criteria	Supportive criteria	Main criteria	Supportive criteria
Sinus tract	o		o		o	
Identical microorganisms isolated form 2 or more cultures	o		o		o	
Purulence surrounding the prosthesis		o	o			
Inflammation in histological examination of prosthetic tissue		o		o		o
Single positive culture		o				o
Single positive culture with virulent microorganism				o		
Elevated synovial fluid leukocyte count		o				o
Elevated synovial fluid neutrophil percentage		o				o
Elevated serum ESR and CRP values		o				o

A total of 79 studies were included, and the data were extracted from the studies. Subsequently, sensitivities, specificities, positive and negative likelihood ratios and positive and negative predictive values were calculated from the extracted data if they were not stated in the publication.

The evidence levels of the selected studies were taken into account, fulfilling the formal requirement of the International Organization for Standardization (ISO) for development of algorithms. The creation of the algorithm was performed according to ISO norm 5807 Modification ITU-I, initially designed for telecommunication defaults, to ensure explicit decision-making criteria for a logical and standardized procedure. The ISO 5807 norm defines the use of a different symbol for the single operation to create an operation plan that has only one input and output [26]. An algorithm that meets the ISO 5807 criteria is composed of process and decision symbols that differ from the symbols for the initial criteria and endpoints. Checklists are introduced to reduce the number of decision symbols. For more practical reasons, the algorithm should not exceed a single page in length.

## Results

**Fig. 2:**
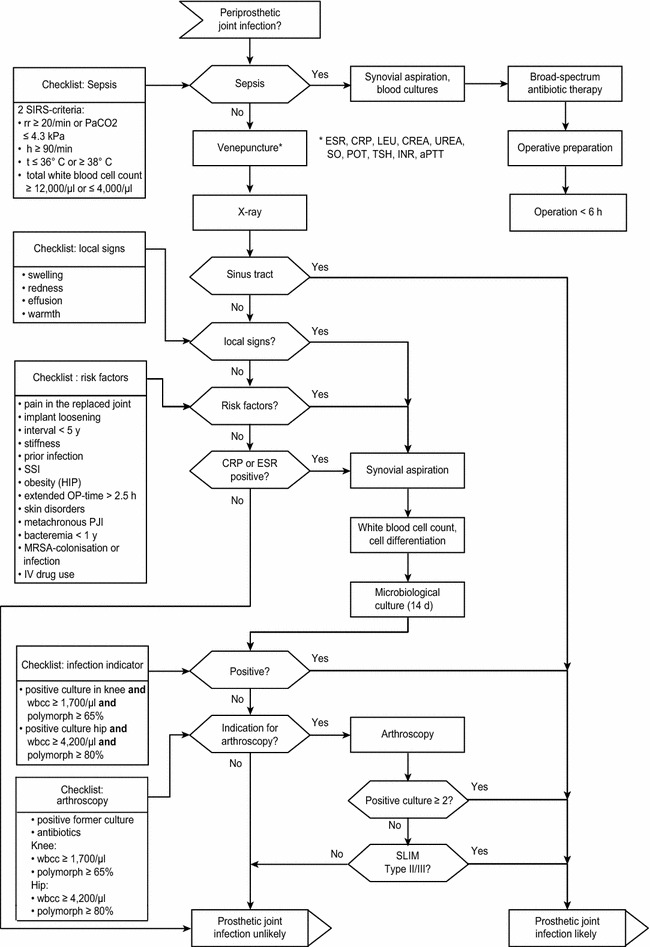
Diagnostic algorithm

The algorithm was a composition of evidence-based procedures developed in our clinic that fulfilled the ISO 5807. Studies were integrated dependent of their LoE in a logical, structured, priority-orientated way in the algorithm. Checklists are located on the left side, the vertical flow represents the main diagnostic criteria and treatment, and the horizontal flow represents the supportive criteria [27] (Fig. 2). For practical reasons, the decisions symbol has been modified to a binary-decision hexagon.

All diagnostic aspects of the algorithm and the underlying literature are specified below.

### Risk factors checklist

In total, 31 included studies (patients: *n* = 312.946; LoE I: *n* = 2, LoE II: *n* = 13, LoE III: *n* = 16) discussed the risk factors for a PJI (Table 3). According to a study by Virolainen, pain in the index joint exhibits a specificity of 100% in patients with PJI. Various consensus recommendations and expert opinions consider a limited range of motion in the total joint an indicator for PJI [25]. The risk factors according to studies with a class of recommendation of I (CoR I) are an extended operation time (*n* = 142.120) [28–32], obesity (*n* = 116,682) [33–41], malnutrition (*n* = 678) [42], diabetes (*n* = 72,778) [35, 39, 40, 43], immunosuppression (n = 86,675) [32, 34, 35, 37, 41, 44, 45], Prior infection of the joint [44], prior infection [44], early implant failure [46], early implant loosening [46, 47] and superficial surgical site infections [32, 48, 49]. Other risk factors have been associated with PJI, although the related studies do not provide strong evidence (CoR II–III); these factors include asymptomatic bacteriuria [50] and tooth interventions, oral surgery and colonoscopies, which provide a crucial risk factor for PJI because of relevant bacteraemia [51, 52]. Likewise, skin disorders in the surgical area during implantation (psoriasis, chronic venous stasis, skin ulcers, lymphedema) increase the risk of implant-associated infection [41, 53].

**Table 3:** Riskfactors for prosthetic joint infections

Author	Year	LoE	(*n* = *x*)	Risk factor	CoR
Huotari et al.	2007	LoE II	*n* = 8201		
Smarbrekke et al.	2004	LoE II	*n* = 31,750		
Kurtz et al.	2010	LoE II	*n* = 69,663	Extended time	CoR I
Uckay et al.	2009	LoE I	*n* = 6001		
Berbari et al.	1998	LoE II	*n* = 26,505		
Dowsey et al.	2009	LoE III	*n* = 1214		
Peersman et al.	2001	LoE III	*n* = 6120		
Lübbeke et al.	2007	LoE II	*n* = 2495		
Dowsey et al.	2008	LoE III	*n* = 1207		
Pulido et al.	2008	LoE III	*n* = 9245	Obesity	CoR I
Namba et al.	2012	LoE II	*n* = 30,491		
Namba et al.	2013	LoE II	*n* = 56,216		
Malinzak et al.	2009	LoE III	*n* = 8494		
Peel et al.	2011	LoE III	*n* = 1200		
Berbari et al.	2012	LoE III	*n* = 678	Malnutrition	CoR III
Namba et al.	2013	LoE II	*n* = 56,216		
Malinzak et al.	2009	LoE III	*n* = 8494	Diabetes	CoR I
Peersman et al.	2001	LoE III	*n* = 6120		
Mraovic et al.	2011	LoE III	*n* = 1948		
Dowsey et al.	2008	LoE III	*n* = 1207		
Peersman et al.	2001	LoE III	*n* = 6120		
Jämsen et al.	2009	LoE II	*n* = 43,149	Immunsuppression	CoR I
Pulido et al.	2008	LoE II	*n* = 9245		
Peel et al.	2011	LoE III	*n* = 63		
Berbari et al.	1998	LoE II	*n* = 26,505		
Bongratz et al.	2008	LoE III	*n* = 462		
Jämsen et al.	2009	LoE II	*n* = 43,149	Prior infection	CoR II
Aslam et al.	2010	LoE III	*n* = 126		
Coelho-Prabhu et al.	2013	LoE III	*n* = 678	Bacteremia	CoR III
Murdoch et al.	2001	LoE III	*n* = 80		
Murray et al.	1991	Level III	*n* = 159	Metachronous infections	CoR III
Luessenhop et al.	1996	Level III	*n* = 145		
Portillo et al.	2013	LoE I	*n* = 116	Implant loosening	CoR I
Sousa et al.	2013	LoE III	*n* = 2497	Bacteriuria	CoR III
Berbari et al.	2010	Level III	*n* = 678	Dental	CoR III

### Sepsis checklist

As a first step, sepsis and septic shock are ruled out. Concerning sepsis, we included 9 studies discussing diagnostic parameters and treatment options for highly acute PJIs. Two LoE I studies, three LoE II studies and six LoE III studies were included. To identify sepsis, we used the diagnostic criteria published by Llewelyn and Dellinger (CoR III) [54–56]. Prior to the initiation of a calculated antibiotic therapy, a synovial aspiration (CoR III) for subsequent examination of cell counts and differentials and a microbiologic workup to identify the causative pathogen should be performed [55, 57, 58]. Additionally, blood cultures should be obtained (CoR I) [59, 60]. Subsequently, early surgical focus management can significantly reduce the mortality rate (CoR II) [61, 62].

### Physical exam

The highest LoE (CoR III) for physical examination is found in a study by Teller et al. [63]. They report a sensitivity of 18.95% (CI 0.05–0.4) and a specificity of 100% (CI 0.98–1.0) for the identification of PJI based on local signs of inflammation, such as warmth, effusion, redness and swelling of the corresponding joint [63]. For fever, a sensitivity of 9% (CI 95 0.03–0.21) and a specificity of 99% (CI 95 0.98–1.00) were reported. These data contribute to a negative predictive value of 0.89 and a negative likelihood ratio of 0.82 (CI 0.66–0.98) for local signs of inflammation (Table 4). Studies with higher levels of evidence for clinical signs of inflammation have not yet been published.

**Table 4:** Physical Exam

Author	Year	LoE	CoR	(*n* = *x*)	Sensitivity	Specificity	Positive predictive value	Negative predictive value	LR for a positive result	LR for a negative result
Teller et al.	2000	III	III	166						
Local signs					0.18 (0.02–0.34)	1	1	0.5 (0.34–0.66)	n.p.	0.82 (0.66–0.98)
Fever					0.9 (0.03–0.21)	0.99 (0.98–1.00)	0.67 (0.13–1.2)	0.88 (0.83–0.93)	13.09 (1.42–31.88)	0.92 (0.78–1.05)

### Inflammatory markers

Decision paths for the CRP and ESR were derived from six studies with a LoE of I, resulting in a CoR I. In this context, Bottner et al. showed significantly increased preoperative CRP and ESR levels in patients with PJI compared with cases with aseptic knee and hip revisions. Considering a cut-off value of 1.5 mg/dl for the CRP, the authors reported a sensitivity of 0.95 (95% CI 0.86–1.0) and a specificity of 0.91 (0.84–0.99). The ESR showed a lower sensitivity of 0.81 (0.64–0.98) and a lower specificity of 0.89 (0.82–0.97) compared with the CRP given a cut-off value of 32 mm/h [64]. In their series of total knee arthroplasties, Valle Della et al. reported sensitivities of 0.95 (0.89–1.0) and 0.9 (0.81–0.99) and specificities of 0.75 (0.64–0.87) and 0.66 (0.53–0.79) for the CRP and ESR, respectively [65]. Greidanus et al. observed a lower sensitivity of 0.82 (0.71–0.95) and a higher specificity of 0.88 (0.76–0.9) for the ESR, with a cut-off value of 30 mm/h. The CRP showed a sensitivity of 0.93 (0.86–1.0) and a specificity of 0.83 (0.76–0.9) given a cut-off value of 1.0 mg/dl [66]. In the 1980s, Kamme et al. determined the ESR and reported a sensitivity of 0.89 (0.8–0.95) and a specificity of 0.73 (0.54–0.9) [67]. Schinsky et al. showed high sensitivity [ESR: 0.96 (0.91–1.0); CRP: 0.95 (0.89–1.0)] and a low specificity [ESR: 0.39 (0.31–0.47); CRP: 0.71 (0.94–1.0)] for the ESR and CRP [68]. In contrast, Savorino et al. reported a low sensitivity [ESR: 0.6 (0.3–0.9); CRP 0.38 (0.14–0.61)] but a higher specificity [ESR: 0.94 (0.82–1.0); CRP: 0.7(0.42–0.98)] [69]. Recently, Fink et al. calculated a sensitivity of 0.73 (0.59–0.86) and a specificity of 0.81 (0.73–0.88) for the CRP in their series of total knee arthroplasties [70]. Positive and negative predictive values and positive and negative likelihoods were calculated and are shown in Table 5. Inflammatory markers such as IL-6, PCT and TNF-alpha have also been the focus of clinical trials; however, there is no Level I or Level II study indicating their superior diagnostic value [71–73].

**Table 5:** Value of serological analysis of c-reactive protein (CRP) and erythrocyte sedimentation rate (ESR)

Author	Year	LoE	CoR	(*n* = *x*)	Sensitivity	Specificity	Positive predictive value	Negative predictive value	LR for a positive result	LR for a negative result
Bottner et al.	2007	I	I	78						
CRP (1.5 mg/dl)					0.95 (0.86–1.0)	0.91 (0.94–0.99)	0.8 (0.64–0.96)	0.98 (0.94–1.0)	10.86 (5.3–73.07)	0.05 (0.0–0.17)
ESR (32 mm/h)					0.81 (0.64–0.98)	0.89 (0.82–0.97)	0.74 (0.56–0.92)	0.93 (0.8–1.0)	7.69 (3.47–38.2)	0.21 (0.02–0.44)
Savarino et al.	2004	I	I	26						
ESR (50 mm/h)					0.6 (0.3–0.9)	0.94 (0.82–1.0)	0.86 (0.71–1.0)	0.79 (0.61–0.97)	9.6 (1.64–16.1)	0.43 (0.09–0.86)
CRP (2 mg/dl)					0.38 (0.14–0.61)	0.7 (0.42–0.98)	0.67 (0.36–0.97)	0.42 (0.18–0.65)	1.25 (0.24–38.34)	0.89 (0.39–2.07)
Kamme et al.	1980	I	I	63						
ESR(30 mm/h)					0.89 (0.8–0.99)	0.72 (0.54–0.9)	0.83 (0.71–0.94)	0.82 (0.66–0.98)	3.2 (1.75–9.54)	0.15 (0.01–0.37)
Greidanus et al.	2007	I	I	151						
ESR (30 mm/h)					0.82 (0.71–0.93)	0.88 (0.81–0.94)	0.74 (0.62–0.86)	0.92 (0.87–0.97)	6.7 (3.84–15.52)	0.2 (0.07–0.36)
CRP (1.0 mg/dl)					0.93 (0.86–1.0)	0.83 (0.76–0.9)	0.7 (0.58–0.82)	0.97 (0.93–1.0)	5.5 (3.57–10.23	0.08 (0.01–0.18)
Della Valle et al.	2007	I	I	94						
ESR (30 mm/h)					0.9 (0.81–0.99)	0.66 (0.53–0.79)	0.67 (0.55–0.8)	0.9 (0.8–0.99)	2.66 (1.74–4.68)	0.15 (0.01–0.35)
CRP (1 mg/dl)					0.95 (0.89–1.00)	0.75 (0.64–0.87)	0.75 (0.63–0.87)	0.95 (0.89–1.0)	3.88 (2.45–7.86)	0.06 (0.02–0.18)
Schinsky et al.	2008	I	I	201						
ESR (30 mm/h)					0.96 (0.91–1.0)	0.39 (0.31–0.47)	0.37 (0.29–0.45)	0.97 (0.92–1.0)	1.58 (1.33–1.91)	0.09 (0.0–0.28)
CRP (1 mg/dl)					0.95 (0.89–1.0)	0.71 (0.94–1.0)	0.55 (0.45–0.65)	0.97 (0.94–1.0)	3.29 (2.45–4.69)	0.08 (0.0–0.18)
Fink et al.	2007	I	I	145						
CRP (1.35 mg/dl)					0.73 (0.59–0.86)	0.81 (0.73–0.88)	0.59 (0.45–0.73)	0.89 (0.82–0.95)	3.81 (2.21–7.48)	0.34 (0.15–0.56)
Bottner et al.	2007	I	I	78	0.95	0.87	0.74	0.98	n.p.	n.p.
IL-6 (>12 pg/ml)					0.43	0.94	0.75	0.85	n.p.	n.p.
TNF-a (>40 nl/ml)					0.33	0.98	0.87	0.8	n.p.	n.p.
PCT (>0.3 ng/ml)										
Di Cesare et al.	2005	III	III	58						
Il-6 (>10 pg/ml)					1	0.95 (0.89–1.0)	0.88 (0.73–1.0)	1	21.5 (9.14–60.85)	0

### Sinus tract

According to the criteria of the MSIS, the ISDA and the International Consensus Meeting, a sinus tract communicating with the prosthesis is a criterion for the presence of a PJI [23, 25, 74]. Two studies dealing with microbiological cultures of patients with a sinus tracts were included; however, a pathogen was not identified in all cases (Table 4). Bogut et al. calculated a sensitivity of 0.82 (0.66–0.98) and a specificity of 1.0 (1.0–1.0; LoE II) [75]. Trampus et al. identified a positive microbiological culture in all cases after sonication and described a sensitivity of 1.0 and a specificity of 1.0 (LoE I) [76].

### Joint aspiration (knee)

For tentative PJI of the knee, five studies (5× LoE I) addressing percutaneous aspiration of synovial fluid were included. Based on an exclusive bacteriologic culture analysis, Fink et al. reported a sensitivity of 0.73 (0.59–0.86) and a specificity of 0.95 (0.91–0.99) [70]. Della Valle et al. reported a similar result for microbiological cultures (sensitivity 0.8 (95% CI n.p.); specificity 0.93 (95% CI n.p.) [65]. Four authors examined the synovial fluid, considering white blood cell counts (WBC) and cell differentiation (Neutrophil-%). Trampuz et al. observed a sensitivity of 0.94 (0.86–1.0) for WBC and of 0.97 (0.91–1.0) for Neutrophil-%, with specificities of 0.88 (0.81–0.94) and 0.98 (0.95–1.0), respectively [77]. Della Valle et al. showed comparable results: WBC sensitivity 0.91 (0.86–0.95)/specificity 1; Neutrophil-% sensitivity 0.98 (0.93–1.0) and specificity 0.85 (0.75–0.95). Ghanem et al. showed a sensitivity for WBC of 0.91 (0.86–0.95) and a specificity of 0.88 (0.84–0.92); for Neutrophil-%, they found a sensitivity of 0.95 (0.92–0.98) and a specificity of 0.95 (0.92–0.97). Zmistowski et al. confirmed the results in their series, reporting a sensitivities of 0.93 (0.87–0.99) for WBC and Neutrophil-% and specificities of 0.94 (0.88–0.99) and 0.83 (0.75–0.91), respectively. Positive and negative predictive values and positive and negative likelihoods were calculated for all studies and are shown in Table 6.

**Table 6:** Value of joint aspiration in the diagnosis of infected total knee arthroplasty

Author	Year	LoE	CoR	(*n* = *x*)	Sensitivity	Specificity	Positive predictive value	Negative predictive value	LR for a positive result	LR for a negative result
Della Valle et al.	2007	I	I	94						
Aspiration culture					0.8	0.93	0.94	0.84	n.p.	n.p.
WBC (3.0 × 10^3^)					0.98 (0.93–1.9)	1	1	0.98 (0.94–1.0)	X	0.02
Neutrophile-%					0.98 (0.93–1.0)	0.85 (0.75–0.95)	0.83 (0.73–0.94)	0.98 (0.94–1.0)	6.46 (3.75–18.75)	0.03 (0–0.1)
Fink et al.	2007	I	I	145						
Aspiration culture					0.73 (0.59–0.86)	0.95 (0.91–0.99)	0.85 (0.73–0.97)	0.9 (0.85–0.96)	15.23 (6.64–125.4)	0.29 (0.14–0.45)
Ghanem et al.	2008	I	I	429						
WBC (3.0 × 10^3^)					0.91 (0.86–0.95)	0.88 (0.84–0.92)	0.82 (0.76–0.88)	0.94 (0.91–0.97)	7.59 (5.45–11.81)	0.11 (0.05–0.16)
Neutorphile (64%)					0.95 (0.92–0.98)	0.95 (0.92–0.97)	0.92 (0.87–0.96)	0.97 (0.95–0.99)	18.19 (11.62–38.43)	0.05 (0.02–0.09)
Trampuz et al.	2007	I	I							
WBC (1.7 × 10^3^)					0.94 (0.86–1.0)	0.88 (0.81–0.94)	0.73 (0.60–0.86)	0.98 (0.95–1.0)	7.76 (4.65–17.92)	0.07 (0.0–0.17)
Neutrophi (65%)					0.97 (0.91–1.0)	0.98 (0.95–1.0)	0.94 (0.87–1.0)	0.99 (0.97–1.0)	48.04 (19.07–136.76)	0.03 (0.0–0.09)
Zmistowski et al.	2012	I	I	150						
WBC (3.0 × 10^3^)					0.93 (0.87–0.99)	0.94 (0.88–0.99)	0.93 (0.87–0.99	0.94(0.88–0.99)	14.35 (7.28–99)	0.07 (0.01–0.14)
Neutrophile (75%)					0.93 (0.87–0.99)	0.83 (0.75–0.91)	0.84 (0.76–0.92)	0.93 (0.87–0.99)	5.52(3.46–11.62)	0.08 (0.01–0.17)

### Joint aspiration (hip)

The diagnostic value of percutaneous synovial aspiration in total hip arthroplasties was addressed in six studies corresponding to a LoE of I. Four studies addressed microbiological cultures, and two studies addressed the WBC and Neutrophil-% of the synovial aspiration (Table 7).

**Table 7:** Value of joint aspiration in the diagnosis of infected total hip arthroplasty

Author	Year	LoE	CoR	(*n* = *x*)	Sensitivity	Specificity	Positive predictive value	Negative predictive value	LR for a positive result	LR for a negative result
Barrack et al.	1993	I	I	291						
Aspiration culture					0.6 (0.3–0.9)	0.88 (0.84–0.92)	0.15 (0.04–0.27)	0.98 (0.97–1.0)	5.11 (1.91–11.32)	0.45 (0.1–0.83)
Mulcahy et al.	1996	I	I	71						
Aspiration culture					0.69 (0.46–0.91)	0.91 (0.83–0.99)	0.69 (0.46–0.91)	0.91 (0.83–0.99)	7.56 (2.76–61.25)	0.34 (0.09–0.65)
Williams et al.	2004	I	I	273						
Aspiration culture					0.8 (0.71–0.9)	0.94 (0.9–0.97)	0.81 (0.72–0.91)	0.93 (0.90–0.97)	12.47 (7.23–29.34)	0.21 (0.11–0.32)
Malhotra et al.	2004	I	I	41						
Aspiration culture					0.44 (0.12–0.77)	0.91 (0.81–1.0)	0.57 (0.2–0.94)	0.85 (0.73–0.97)	4.74 (0.62–106.19)	0.61 (0.23–1.09)
Schinsky et al.	2008	I	I	201						
Wbc (4.2 × 10^3^)					0.84 (0.74–0.93)	0.93 (0.89–0.97)	0.82 (0.72–0.92)	0.94 (0.90–0.98)	12.21 (6.75–33.94)	0.18 (0.07–0.29)
Neutrophile (80%)					0.82 (0.72–0.92)	0.83 (0.77–0.89)	0.64 (0.53–0.76)	0.92 (0.88–0.97)	4.78 (3.08–8.36)	0.22 (0.09–0.37)
Spanghel et al.	1999	I	I	183						
WBC (5.0 × 10^3^)					0.36 (0.18–0.53)	0.99 (0.98–1.0)	0.91 (0.74–1.0)	0.9 (0.85–0.94)	55.36 (9.43–86.89)	0.65 (0.46–0.84)
Neutrophile (80%)					0.89 (0.78–1.0)	0.85 (079–0.91)	0.52 (0.38–0.66)	0.98 (0.95–1.0)	5.94 (3.76–10.75)	0.13 (0.0–0.28)
Dinneen et al.	2013	I	I	75						
WBC (1.58 × 10^3^)					0.895 (0.783–0.997)	0.913 (0.827–0.999)	n.p.	n.p.	n.p.	n.p.
Neutrophile (65%)					0.897 (0.795–0.999)	0.866 (0.761–0.971)	n.p.	n.p.	n.p.	n.p.

Mulcahy et al. reported a sensitivity of 0.69 (0.46–0.91) and a specificity of 0.91 (0.83–0.99) for microbiological culture [78]. Similar results with a sensitivity of 0.44 (0.12–0.77) and a specificity of 0.91 (0.81–1.0) were published by Malhotra et al. [79], and Barrack et al. reported a sensitivity of 0.6 (0.3–0.9) and a specificity of 0.88 (0.84–0.92) [80]. Williams et al. reported a higher sensitivity of 0.8 (0.71–0.9) and a specificity of 0.94 (0.9–0.97) [81], while Schinsky et al. published a sensitivity of 0.84 (0.74–0.93) and specificity of 0.93 (0.89–0.97) for cell count analysis and a sensitivity of 0.82 (0.72–0.92) and specificity 0.83 (0.77–0.89) [68]. The results confirm the series of Dinneen et al., who reported a sensitivity of 0.89 (0.783–0.997) and specificity of 0.91 (0.827–0.99); for WBC, the values were 0.89 (0.79–0.99) and 0.86 (0.76–0.97), respectively [82].

Other synovial fluid markers, such as synovial CRP and synovial IL-6, and antimicrobial peptides, such as alpha-defensin, are undergoing clinical trials [6, 83–85]. However, there is no Level I or Level II study indicating their superior diagnostic value.

### Synovial biopsy histological workup

Overall, seven LoE I studies including 822 patients (5× frozen sections/2× fixed sections) addressing synovial biopsy and histological workup and one LoE III study establishing a histopathological classification of the periprosthetic membrane were included (Table 8). Banit et al. showed a sensitivity of 0.45 (0.16–0.75) and specificity of 0.92 (0.85–1.0) in their cohort. Borrego et al. reported a sensitivity of 0.5 (0.15–0.85), and a specificity of 1.00 in their series of 83 patients with THA and a sensitivity of 0.67 (0.48–0.86) and a specificity of 0.8 (0.7–0.93) in their series of 63 patients with TKA [86].

**Table 8:** Value of synovial biopsy

Author	Year	LoE	CoR	(*n* = *x*)	Sensitivity	Specifity	Positive predictive value	Negative predictive value	LR for a positive result	LR for a negative result
Banit et al.	2002	I	I							
Frozen section (Hip)				63	0.45 (0.16–0.75)	0.92 (0.85–1.0)	0.56 (0.23–0.88)	0.89 (0.81–0.97)	5.91 (1.07–166.5)	0.59 (0.25–0.99)
Frozen section (knee)				55	1	0.96 (0.9–1.0)	0.82 (0.59–1.0)	1	23.00 (9.76–64.7)	0
(Knee + hip.shoulder)				121	0.67 (0.48–0.86)	0.95 (0.91–1.0)	0.8 (0.62–0.98)	0.91 (0.86–0.97)	14.67 (5.37–442)	0.35 (0.15–0.57)
Borrego et al.	2007	I	I							
Frozen sectio (Hip)				83	0.5 (0.15–0.85)	1	1	0.95 (0.9–1.0)	X	0.5 (0.15–0.85)
Frozen section (knee)				63	0.67 (0.48–0.86)	0.9 (0.8–0.99)	0.8 (0.62–0.98)	0.81 (0.7–0.93)	6.5 (2.42–116.4)	0.37 (0.15–0.65)
Schinsky et al.	2008	I	I	201						
Frozen section					0.73 (0.61–0.84)	0.94 (0.9–0.98)	0.82 (0.71–0.92)	0.9 (0.85–0.95)	11.8 (6.06–37.34)	0.29 (0.16–0.43)
Fink et al.	2008	I	I	145						
Fixed section (knee)					1	0.98 (0.95–1.0)	0.95 (0.89–1.0)	1	52.5 (22.13–140.88)	X
Tissue culture (knee)					0.78 (0.65–0.9)	0.98 (0.95–1.0)	0.94 (0.86–1.0)	0.92 (0.87–0.97)	40.69 (14.28–127.41)	0.23 (0.09–0.37)
Both					0.78 (0.65–0.9)	0.98 (0.95–1.0)	0.94 (0.86–1.0)	0.92 (0.87–0.97)	40.69 (14.28–127.41)	0.23 (0.09–0.37)
Della Valle et al.	2007	I	I	94						
Forzen section					0.88 (0.78–0.98)	0.96 (0.91–1.0)	0.95 (0.88–1.0)	0.91 (0.84–0.99)	23.27 (8.74–72.1)	0.13 (0.02–0.24)
Fink et al.	2013	I	I	100						
Fixed section (hip)					0.62 (0.48–0.76)	1	1	0.76 (0.67–0.86)	X	0.38 (0.24–0.52)
Tissue culture (hip)					0.73 (0.6–0.86)	0.98 (0.95–1.0)	0.97 (0.91–1.0)	0.82 (0.73–0.91)	40.33 (11.29–50.36)	0.27 (0.14–0.42)
Both					0.82 (0.71–0.93)	0.98 (0.95–1.0)	0.97 (0.92–1.0)	0.87 (0.06–0.31)	45.22 (13.28–54.52)	0.18 (0.06–0.31)

Nunez et al. calculated a sensitivity of 0.86 (0.76–0.96) and a specificity of 0.87 (0.8–0.94) [87].

Banit et al. observed a sensitivity of 1.0 and a specificity of 0.96 (0.9–1.0) for TKA and a sensitivity of 0.45 (0.16–0.75) and a specificity of 0.92 (0.85–1.0) for THA. Overall, the authors reported a sensitivity of 0.67 (0.48–0.86) and a specificity of 0.95 (0.91–1.0) [88]. In their series of 105 patients with painful TKA, Valle Della et al. observed a sensitivity of 0.88 (0.78–0.98) and a specificity of 0.96 (0.91–1.0). For THA, Schinsky et al. reported a sensitivity of 0.73 (0.61–0.84) and a specificity of 0.94 (0.9–0.98) [65, 68].

Regarding fixed sections, Fink et al. showed a sensitivity of 1.0 and a specificity of 0.98 (0.95–1.0) for TKA (145) and a sensitivity of 0.62 (0.48–0.76) and a specificity of 1.0 for THA (100) [70, 89].

### Synovial biopsy microbiological workup

In their series of THAs, Fink et al. showed a sensitivity of 0.73 (0.6–0.86) and a specificity of 0.98 for microbiological culture of biopsies (0.95–1.0). The combination of histological biopsy and microbiological culture increased the sensitivity to 0.82 (0.71–0.93) [90]. In their series of TKAs, they reported a sensitivity of 0.78 (0.65–0.9) and a specificity of 0.98 (0.95–1.0); when combined with histological analysis, the sensitivity increased to 1, with equal specificity. Regarding the recommended number of microbiological cultures, the mathematical model of Atkins et al. was used: at least 5 or 6 biopsy cultures should be taken [91]. Marin et al. showed a sensitivity of 0.87 (0.66–0.94) and a specificity of 0.67 (0.56–0.76) for one positive microbiological culture using that model; when three positive cultures were used, the sensitivity decreased to 0.46 (0.32–0.61), while the specificity increased to 0.98 (0.93–0.99) [92].

## Discussion

Although the diagnosis of PJIs prior to revision surgery is of paramount importance for further treatment, it can be challenging, and a well-structured diagnostic approach is necessary. A PJI diagnosis results in substantial changes in the therapeutic procedure [3]. Thus, an evidence-based and priority-orientated algorithm (Fig. 2) can provide an incremental and easy-to-use guideline for non-specialists and less-experienced orthopedic surgeons.

AAOS guidelines strongly recommend determining the ESR and CRP [93]. According to the included studies, sensitivities vary from 81 to 93% for the ESR and from 73 to 95% for the CRP [93]. In a recent meta-analysis by Berbari and colleagues that included 30 studies with a total of 3909 patients, pooled sensitivities of 75% for the ESR and 88% for the CRP were reported [94]. The specificities were 70 and 74%, respectively. Despite the relatively high sensitivity of the CRP, its specificity remains unsatisfactory, confirming the observations of McArthur et al. who identified a considerable subset of patients with PJI and negative serology within their series of 414 infected THAs. In contrast, the AAOS guidelines recommend percutaneous aspiration only in case of altered ESR and CRP levels and thus exclude seronegative patients from this procedure [93]. In these cases, one-stage revision surgery without adequate antibiotic treatment may be performed, inevitably resulting in new prosthetic failure and PJI persistence. In our algorithm, the decision to perform joint aspiration is based on ESR and CRP levels and on the radiological findings and medical history (risk factors) of the patient.

Large multicenter LoE I studies were able to define some risk factors. In particular, potential intraoperative contamination and the immune system of the patients were determined to have an important role. Namba et al. showed in a large multicenter study that an extended operation time leads to an increase of PJIs. An additional 15 min of operation time was determined to increase the risk by up to 9% [39]. This relationship is explained by increased time for potential intraoperative microbial contamination. However, the increased risk of PJI in immunocompromised patients, such as those with rheumatoid arthritis and/or diabetes, has also been proven. The most recent studies suggest that even asymptomatic bacteriuria is an independent risk factor for PJI; the authors indicated that an immunocompromised status puts patients at risk for colonization with Gram-negative microorganisms [50]. Early implant loosening (<5 years) without evidence of mechanical failure or progressive radiolucency adjacent to the implant must be considered a decisive risk factor for a low-grade PJI. As proposed by Lachiewicz et al., premature implant loosening and the presence of the previously described risk factors require further diagnostic procedures [47]. In this context, Portillo et al. were able to demonstrate a significantly longer period between primary implantation and diagnosed aseptic loosening (7.8 years) compared with septic implant loosening (2 years; CoR I) [46].

Considering the aforementioned evidence-based risk factors in our algorithm prior to joint aspiration permits a benefit-risk assessment for post-interventional complications and economic issues. Although iatrogenic complications in the context of synovial aspiration are considered rare, Murray et al. reported a complication rate of 5.1% (0.2–10%), including hematoma, infections and lesions on nerve structures after synovial aspiration of the hip [95]. Barrack et al. showed a 1% (0.1–2.2%) rate of infections after synovial aspiration of the hip [96]. This benefit–risk assessment is of major importance to minimize the risk of infection for the patient and thus avoid false-positive results leading to overtreatment [97].

However, the diagnostic value of synovial aspiration and subsequent microbiological workup is controversial according to recent literature. Sensitivities vary between 12 and 89%, with specificities between 50 and 100% for synovial aspiration of hip joints [68, 78, 79, 81, 82, 96, 98, 99]. Similar results are available for TKA [65, 70, 77, 100, 101]. However, extended synovial analysis combining microbiological culture with WBC and neutrophil-% is the gold standard for synovial aspiration investigation. The sensitivity, specificity, positive/negative predictive value and positive/negative likelihood ratio for the WBC and neutrophil-% are given in Tables 6 and 7. Several studies have examined the optimal cut-off values for WBC and neutrophil-%. Trampuz et al. suggest 1.7 × 10^3^/µl (WBC) and 65% (neutrophil-%), and Zmistowski et al. and Della Valle report quite similar results, using higher cut-off values of 3.0 × 10^3^ for WBC and 75% neutrophils [65, 101]. However, cut-off values calculated using receiver-operating characteristics are linked to the microbiological strains that cause the PJI. In our algorithm, we used the lower cut-off values that Trampuz et al. and Schinsky et al. identified to ensure that we detected the low-grade infections caused by slow-growing and low virulence strains, such as coagulase-negative *staphylococci* or *Proprionibacterium acnes*, which generate a low immune reaction. Considering the defining criteria for PJI (Table 2), 2 or more separate synovial fluid samples should be obtained from the index joint. However, this main criterion is usually not met with routine synovial aspiration. In the daily clinical routine, the additional use of blood culture bottles, as proposed by Minassian et al. [102], to obtain two separate microbiological cultures should thus be encouraged. Although the data on diagnostic value are discordant, the causative pathogen and its antibiotic sensitivity pattern can be identified via synovial aspiration and microbiological examination. This information, in turn, is of great importance for preoperatively planning the surgical strategy and the antibiotic regimen.

Unfortunately, a causative pathogen can only be identified in approximately 44% [79]–80% [81] of cases, reflecting the heterogeneous diagnostic value of synovial aspiration. Among the factors influencing microbiological results, the length of the incubation period is crucial because the bacteria that cause PJIs occur only in a very low number in the biofilm and often are in a sessile form that is very slow growing [103, 104]. Accordingly, in many of the aforementioned studies, the length of microbiological incubation was only 48 h or was not specifically disclosed. Furthermore, the omission of antibiotic treatment termination at least 2 weeks prior to the joint aspiration can lead to false-negative microbiological results and thus to maltreatment [105].

Other synovial fluid markers, such as alpha-defensin, show promising results, with a sensitivity of 100% [4, 85], but they lack the evidence and independent studies to support their use. Similarly, the leukocyte esterase test requires further evidence to support its role in diagnosing PJI [5, 106]. According to the Plan-Do-Check-Act principle, constant improvement of the algorithm by reintegrating actual evidence-based literature at half-year intervals is intended. If new diagnostic procedures fulfill the LoR I criteria, they will be included in the algorithm.

As a further, more invasive diagnostic step, arthroscopic synovial biopsy has been implemented in our algorithm. As itemized in the “Arthroscopy” checkbox (Fig. 2), increased WBC or neutrophil percentage but negative microbiological assessment of the aspirate, continued antibiotic treatment and history of PJI are indications for synovial biopsy according to our algorithm.

In this context, recent studies by Williams et al. showed equal results for aspiration and tissue biopsy with sole microbiological examination [81]. These results underline the importance of concurrent histological and microbiological workup of the biopsy specimens, as confirmed by Malhotra and Morgan in their series of 41 THAs [79]. The authors reported a sensitivity of 80% and a specificity of 100% for synovial biopsy compared with a sensitivity of 44% and a specificity of 91% for synovial fluid aspiration. Likewise, Fink et al. reported synovial biopsy sensitivities of 100 and 87% for TKA and THA, respectively [70, 89] Specificity was 98% for both TKA and THA. According to the authors, the underlying hypothesis for the discrepancy of results between hip and knee joints was that biopsy samples can be obtained at many more places adjacent to the prosthesis in the knee compared with hip joints, where only the head and neck of the prosthesis and the inlay of the acetabular cup are easily accessible [89].

Despite its excellent diagnostic value, synovial biopsy should only be applied in selected cases, as stated above. Although the procedure can be considered a minor operation, potential risks such as neuro-vascular injury or surgical site infection should not be underestimated [107]. Furthermore, arthroscopic instruments can damage the hip or knee replacement, particularly in ceramic implants. Thus, similar to percutaneous joint aspiration, a benefit–risk assessment prior to the intervention is essential to maximize the diagnostic yield within the diagnostic cascade and while minimizing the potentially harmful effects for the patient.

## Conclusions

The diagnostic algorithm presented in this study is derived from high-quality studies in the field of PJI and provides a well-structured diagnostic approach in form of a detailed and transparent SOP. These incremental and easy-to-use guidelines facilitate consistently high and examiner-independent process quality in terms of PJI treatment and provide a basis for scientific analyses.

## Abbreviations

PJI: prosthetic joint infection
THA: total hip arthroplasty
TKA: total knee arthroplasty
EAST: Eastern Association of the Surgery of Trauma
SOP: standard operating procedure
ESR: erythrocyte sedimentation rate
CRP: c-reactive protein
GRADE: The Grading of Recommendation Assessment, Development and Evalutation
STARD: Standards for Reporting of Diagnostic Accuracy
LoE: level of evidence
CoR: class of recommendation
IDSA: Infectious Deceases Society of America
MSIS: Musculoskeletal Infection Society
ISO: International Organization for Standardization
IL-6: interleukin-6
PCT: procalcitonin
AAOS: American Academy of Orthopaedic Surgeons
